# Supplementary material for: Influence of Comprehensive Pre-Anaesthetic Assessment on ASA Classification and Surgical Cancellations in Dogs and Cats: A Retrospective Observational Study
Source: Vet Sci. 2025 Jun 23;12(7):612. doi: 10.3390/vetsci12070612 (PMC12298058; doi:10.3390/vetsci12070612)
Supplement: Supplementary file 1 [file vetsci-12-00612-s001.zip › Pre-anaesthetic Assessment Form.pdf]

CEU Universidad Cardenal Herrera

# FICHA EVALUACIÓN PREANESTÉSICA
## HCV UCH - SERVICIO DE ANESTESIA

CASO FECHA MOTIVO URGENCIA ☐

NOMBRE

**RESEÑA**

ESPECIE perro ◯ gato ◯ otro ◯ RAZA EDAD PESO

SEXO M ◯ H ◯ castrado ☐ CARÁCTER tranquilo ◯ agresivo ◯ nervioso ◯ excitable ◯

ALIMENTACIÓN pienso seco ☐ latas ☐ casera ☐ huesos ☐ médico ☐ otro ☐

APTITUD compañía ◯ guarda ◯ deporte ◯ caza ◯ OTROS ANIMALES ☐

HABITAT piso ◯ chalet ◯ reala ◯ protectora ◯ calle ◯ encontrado ◯ ZONA

**HISTORIA CLÍNICA**

VACUNACIONES PERRO tetra ☐ hepta ☐ rabia ☐ GATO trivalente ☐ leucemia ☐

DESPARASITACIONES pastillas ☐ collar antipulgas ☐ pipetas ☐

PARTOS sí ◯ no ◯ NORMALES sí ◯ no ◯ ÚLTIMO PARTO CELO

CUBIERTA sí ◯ no ◯

**ENFERMEDADES ANTERIORES**

| fecha | diagnóstico | tratamiento | alta |
|---|---|---|---|
| | | | ☐ ☐ |
| | | | ☐ |

**TRATAMIENTOS ACTUALES**

**ANESTESIAS ANTERIORES**

| fecha | protocolo | complicaciones |
|---|---|---|
| | | |

**SIGNOS CLÍNICOS ACTUALES**

alt. peso ☐ alt. comportamiento ☐ dolor ☐ epilepsia ☐ sec. nasal ☐ disnea ☐ tos ☐

síncopes ☐ epistaxis ☐ hipersalivación ☐ anorexia ☐ vómitos ☐ diarrea ☐ estreñim ☐

sec. vaginal ☐ quemaduras ☐ edemas ☐ ascitis ☐ PU/PD ☐ disuria/anuria ☐

heridas ☐ tumores ☐ cojera ☐ debilidad ☐

**EXPLORACIÓN FÍSICA**

Actitud general alerta ◯ deprimido ◯ letárgico ◯ postrado ◯ coma ◯

Estado corporal normal ◯ delgado ◯ caquéctico ◯ semiobeso ◯ obeso ◯

Pulso fuerte normal ◯ débil ◯ filiforme ◯ impalpable ◯ déficit ◯

Mucosas normal ◯ pálidas ◯ congestivas ◯ ictéricas ◯ cianóticas ◯

Ausc. cardiaca normal ☐

Ausc. pulmonar normal ☐

Piel normal ☐

Palpación abdominal normal ☐

Ganglios normal ☐

Boca normal ☐

Sistema nervioso normal ☐

Aparato locomotor normal ☐

FC

FR

Tª

%HID

TRC

PAM

PAS

PAD

**CLASIFICACIÓN ASA** I ◯ II ◯ III ◯ IV ◯ V ◯ E ☐ **REALIZADA**

**PRUEBAS COMPLEMENTARIAS**

Hematología sí ◯ no ◯ fecha normal ☐

Bioquímica sérica sí ◯ no ◯ fecha normal ☐

Pruebas de coagulación sí ◯ no ◯ fecha normal ☐

Urianálisis sí ◯ no ◯ fecha normal ☐

Electrocardiograma sí ◯ no ◯ fecha normal ☐

Radiología sí ◯ no ◯ fecha normal ☐

Otros

**CLASIFICACIÓN ASA** I ◯ II ◯ III ◯ IV ◯ V ◯ E ☐ **REVISADA**

## PROTOCOLO ANESTÉSICO PROPUESTO

| | PREM | IND | MAN | POST | | PREM | IND | MAN | POST |
|---|---|---|---|---|---|---|---|---|---|
| **ACP** | ☐ | | ☐ | ☐ | **MRF** | ☐ | | ☐ | ☐ |
| **MED** | ☐ | | ☐ | ☐ | **MTD** | ☐ | | ☐ | ☐ |
| **DEX** | ☐ | | ☐ | ☐ | **PET** | ☐ | | ☐ | ☐ |
| **MDZ** | ☐ | ☐ | ☐ | ☐ | **FEN** | ☐ | ☐ | ☐ | ☐ |
| **DZP** | ☐ | ☐ | ☐ | ☐ | **RMF** | ☐ | ☐ | ☐ | ☐ |
| **PROP** | | ☐ | ☐ | ☐ | **BUP** | ☐ | | ☐ | ☐ |
| **AFX** | ☐ | ☐ | ☐ | ☐ | **BTF** | ☐ | | ☐ | ☐ |
| **TIOP** | | ☐ | ☐ | | **TRAM** | ☐ | | ☐ | ☐ |
| **KET** | ☐ | ☐ | ☐ | ☐ | **MLX** | ☐ | | | ☐ |
| **ETOM** | | ☐ | ☐ | | **CRP** | ☐ | | | ☐ |
| **ISO** | | ☐ | ☐ | | **COXIB** | ☐ | | | ☐ |
| **SEVO** | | ☐ | ☐ | | **LIDO** | ☐ | ☐ | ☐ | ☐ |
| **DESF** | | ☐ | ☐ | | | ☐ | ☐ | ☐ | ☐ |

LOCORREGIONAL No ○ Sí ○ TÉCNICA Epidural ☐ Bloqueo ☐ Descripción

Lido. ☐ Bupi. ☐ Ropi. ☐ Morf. ☐ Otro ☐

FLUIDOTERAPIA No ○ Sí ○ SSF ☐ RL ☐ GS ☐ EB ☐ Coloide ☐ otro ☐

$O_2$ Sí ○ No ○ AIRE Sí ○ No ○ INTUBACIÓN Sí ○ No ○ CIRCUITO Circular ○ T Ayre ○

VENTILACIÓN MECÁNICA No ○ Sí ○ BNM ☐

## CONSENTIMIENTO INFORMADO

**FIRMADO** sí ○ no ○ pendiente ○ no procede ○

Reset Enviar
